# Chronic Dialysis Patients Are Depleted of Creatine: Review and Rationale for Intradialytic Creatine Supplementation

**DOI:** 10.3390/nu13082709

**Published:** 2021-08-06

**Authors:** Yvonne van der Veen, Adrian Post, Daan Kremer, Christa A. Koops, Erik Marsman, Theo Y. Jerôme Appeldoorn, Daan J. Touw, Ralf Westerhuis, Margaretha Rebecca Heiner-Fokkema, Casper F. M. Franssen, Theo Wallimann, Stephan J. L. Bakker

**Affiliations:** 1 Department of Internal Medicine, University Medical Center Groningen, University of Groningen, 9713 GZ Groningen, The Netherlands; d.kremer@umcg.nl (D.K.); c.f.m.franssen@umcg.nl (C.F.M.F.); 2 Department of Laboratory Medicine, University Medical Center Groningen, University of Groningen, 9713 GZ Groningen, The Netherlands; c.a.koops@umcg.nl (C.A.K.); m.r.heiner@umcg.nl (M.R.H.-F.); 3 Dialysis Center Groningen, 9713 GZ Groningen, The Netherlands; e.marsman@dcg.nl (E.M.); r.westerhuis@dcg.nl (R.W.); 4 Department of Clinical Pharmacy and Pharmacology, University Medical Center Groningen, University of Groningen, 9713 GZ Groningen, The Netherlands; t.y.j.appeldoorn@umcg.nl (T.Y.J.A.); d.j.touw@umcg.nl (D.J.T.); 5 Department of Biology, ETH Zurich, 8092 Zurich, Switzerland; theo.wallimann@cell.biol.ethz.ch

**Keywords:** creatine, intradialytic creatine supplementation, hemodialysis, muscle, protein energy wasting, clinical trial, muscle weakness, chronic fatigue, cognitive impairment, depression, anemia

## Abstract

There is great need for the identification of new, potentially modifiable risk factors for the poor health-related quality of life (HRQoL) and of the excess risk of mortality in dialysis-dependent chronic kidney disease patients. Creatine is an essential contributor to cellular energy homeostasis, yet, on a daily basis, 1.6–1.7% of the total creatine pool is non-enzymatically degraded to creatinine and subsequently lost via urinary excretion, thereby necessitating a continuous supply of new creatine in order to remain in steady-state. Because of an insufficient ability to synthesize creatine, unopposed losses to the dialysis fluid, and insufficient intake due to dietary recommendations that are increasingly steered towards more plant-based diets, hemodialysis patients are prone to creatine deficiency, and may benefit from creatine supplementation. To avoid problems with compliance and fluid balance, and, furthermore, to prevent intradialytic losses of creatine to the dialysate, we aim to investigate the potential of intradialytic creatine supplementation in improving outcomes. Given the known physiological effects of creatine, intradialytic creatine supplementation may help to maintain creatine homeostasis among dialysis-dependent chronic kidney disease patients, and consequently improve muscle status, nutritional status, neurocognitive status, HRQoL. Additionally, we describe the rationale and design for a block-randomized, double-blind, placebo-controlled pilot study. The aim of the pilot study is to explore the creatine uptake in the circulation and tissues following different creatine supplementation dosages.

## 1. Introduction

End-stage kidney disease (ESKD) is one of the world’s leading causes of morbidity and mortality. It is estimated that more than 2.5 million people received kidney replacement therapy worldwide in 2010, and this number is projected to increase to over 5 million by 2030 [1]. Dialysis is a life-saving treatment, but unfortunately, the health-related quality of life (HRQoL) of dialysis patients is poor and mortality risks are high, as compared with the general population [2]. Although several potentially modifiable risk factors (e.g., pre-dialysis care and nutritional status) and unmodifiable risk factors (e.g., age and genetics) for excess risk of mortality and poor HRQoL have been identified in dialysis-dependent CKD patients, there is great need for the identification of new, potentially modifiable risk factors [2,3,4,5,6,7]. We hypothesize that creatine deficiency is such a modifiable risk factor, which underlies several important causes for impaired HRQoL in patients with dialysis-dependent chronic kidney disease (CKD), including protein energy wasting (PEW), sarcopenia, fatigue, muscle weakness, depression, cognitive impairment, and increased susceptibility, as well as a higher risk of an adverse course of infectious diseases.

Creatine is a natural nitrogenous organic acid that is integral to energy metabolism, and is crucial for proper cell functioning [8,9]. Creatine can be charged to the high-energy product phosphocreatine by creatine kinase and ATP [4,10]. In the human body, the majority of creatine (>90%) is present in skeletal muscle, cardiac muscle, smooth muscle, the brain, and in the nervous tissue [8,9]. Furthermore, smaller amounts are present in other tissues and cell types, including the kidney, erythrocytes, and leucocytes [8,9]. In all these tissues and cells, the phosphocreatine–creatine circuit serves as an energy buffer, facilitating quick transitions in energy requirements [9]. Importantly, on a daily basis, approximately 1.6–1.7% of the total, mainly intracellular, creatine pool is non-enzymatically degraded to creatinine, which subsequently leaves the cells and is excreted by the kidney as a waste product in urine [11,12]. This loss necessitates a continuous replenishment of the total creatine pool, with new creatine to remain in balance [12].

**Figure 1:**
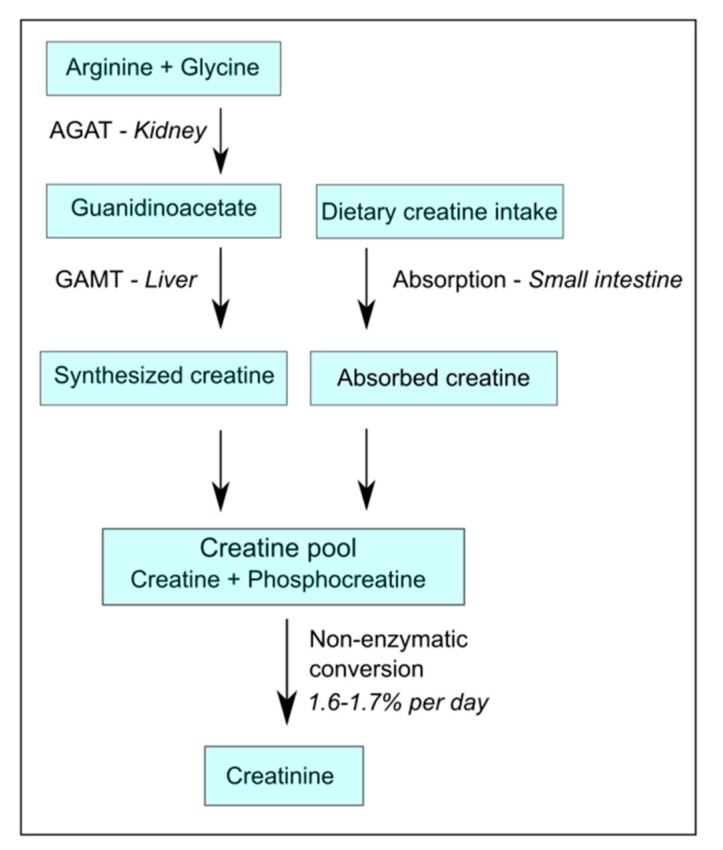
Simplified schematic overview of creatine homeostasis. AGAT-arginine:glycine amidino-transferase; GAMT-guanidinoacetate methyltransferase.

Generally, a common omnivorous diet can provide up to 50% of the daily requirement of creatine replenishment from alimentary sources, like meat, fish, and dairy products [7,12]. Coverage of the other 50% requires endogenous synthesis, and the requirements for endogenous synthesis increase if diets become more plant-based. The endogenous synthesis of creatine involves a metabolic pathway, of which the first and rate-limiting step is primarily situated in the kidney [13], where the enzyme arginine:glycine amidino-transferase (AGAT) converts arginine plus glycine into the creatine precursor guanidinoacetate (GAA) [14], which is exported from the kidney into circulation [8,12]. From there, GAA is taken up into the liver where it is subsequently converted by the enzyme guanidinoacetate methyltransferase (GAMT) into creatine, and is released into the blood stream (Figure 1) [4,8,12]. Because the rate-limiting step of endogenous creatine synthesis takes place in the kidney, and the capacity for the endogenous synthesis of metabolites by AGAT has been shown to be almost halved after the donation of a kidney [7], it may be expected that patients with dialysis-dependent CKD with heavily impaired kidney function would also have a virtually absent capacity for endogenous creatine synthesis [4,7].

It should also be realized that, apart from decreased or absent endogenous synthesis, creatine losses are likely higher in patients with dialysis-dependent CKD than in healthy subjects. In healthy subjects, creatine losses will mainly be limited to the non-enzymatic conversion of creatine to creatinine, as the majority of creatine is reabsorbed after glomerular filtration [15,16,17]. In patients with dialysis-dependent CKD, creatine losses will be the consequence of the non-enzymatic conversion of creatine to creatinine and—on top of that—of unopposed losses into the dialysate, because the dialysis filter cannot reabsorb creatine after filtration from circulation [18]. The same holds true for amino acids, including the AGAT substrates glycine and arginine, which are essential for the synthesis of the creatine precursor GAA, and other valuable small water soluble molecules, like GAA itself [17,18].

The existing tendency towards a negative creatine balance in chronic dialysis patients is further exacerbated by the current dietary recommendations for patients with CKD, which are increasingly steered towards more plant-based diets [19,20]. The reasons for this are that plant-based diets provide lower loads of phosphorus and acid than animal-based diets, which may seem helpful for controlling hyperphosphatemia and acidosis. However, a plant-based diet might put patients with dialysis-dependent CKD at an even higher risk for a negative creatine balance and developing creatine deficiency, because plant-based diets lack naturally derived creatine [21]. It should be noted that amino acids necessary for the endogenous synthesis of creatine are present in plant-based foods, but because of the absence of kidney function in patients with dialysis-dependent CKD, the enzymatic capacity for the endogenous synthesis of creatine from these amino acids is severely impaired [4,7]. This is underscored by the fact that even without dietary recommendations toward a plant-based diet, roughly 43% of the general U.S. population has an average intake of creatine below the recommended daily allowance of 1.0 g of dietary creatine per day [22].

Together, the poor endogenous synthesis of creatine, unopposed loss of creatine and creatine precursors during dialysis, and low dietary intake of creatine may add up to creatine deficiency in patients with dialysis-dependent CKD. In general, the plasma concentrations of small molecules tend to be higher in hemodialysis patients compared with healthy individuals, because of a lower glomerular filtration rate. However, the fasting plasma creatine and GAA levels are lower in dialysis patients compared with healthy individuals [18]. This indicates that dialysis-dependent CKD, in comparison with normal subjects, have a generally lower level of creatine and its metabolites. This is supported by the fact that skeletal muscle biopsies in CKD showed significantly low ATP and phosphocreatine levels [23]. This notion is further supported by studies showing lower phosphocreatine concentrations and a decreased phosphocreatine/ATP energy-charge ratio in the hearts and skeletal muscle of patients on either hemodialysis or peritoneal dialysis treatment, as shown by means of non-invasive in vivo 31P-NMR imaging [24,25,26].

## 2. Rationale

Creatine supplementation is frequently used among athletes, during rehabilitation, and in patients with neuromuscular diseases [27,28,29]. We propose that creatine supplementation is particularly important for patients with dialysis-dependent CKD. The reasons why this is likely to be of particular importance in these patients are because (1) the endogenous synthesis of creatine in these patients is severely impaired because of the virtual absence of kidney function accompanied by the virtual absence of the first enzymatic step required for the endogenous absence of creatine from the amino acids arginine and glycine [4,7]; (2) unopposed losses of creatine to the dialysis fluid during dialysis sessions [17,18]; and (3) inadequate intake of creatine due to advice towards a primary plant-based diet in these patients [21]. All of this comes on top of the normally existing continuous non-enzymatic conversion of approximately 1.6–1.7% of the endogenous creatine pool to creatinine, which necessitates the continuous replenishment of creatine by the combination of endogenous synthesis and dietary intake in order to remain in steady-state [11,12]. This is novel because, until recently, it was not recognized that kidney function is an important contributor to endogenous creatine synthesis, so the capacity of patients with dialysis-dependent CKD to maintain creatine homeostasis in the light of ongoing conversion of creatine into creatinine is severely impaired, and there are additional unopposed losses of creatine to the dialysis fluid and an inadequate dietary intake. Patients with dialysis-dependent CKD could benefit from creatine supplementation by allowing for the maintenance of their endogenous creatine pools, which would help them to sustain bodily functions that depend on creatine availability, including normal functioning of the muscles, heart, immune system, and brain [24,30,31].

Thus far, only a few creatine supplementation trials have been performed in patients with dialysis-dependent CKD. In a double-blind, randomized, placebo-controlled trial with 10 patients, presenting with frequent muscle cramps during hemodialysis, an oral dose of 12 g of creatine monohydrate or placebo was given before each dialysis session for 4 weeks [32]. The authors found a 60% reduction in muscle cramps after four weeks of creatine supplementation, while no difference was found in the placebo group. After a wash-out period, the frequency of muscle cramps returned to the previous level. In another double-blind, randomized, placebo-controlled trial with 30 hemodialysis patients, an initial loading phase of 1 week, in which an oral dose of 40 g of creatine monohydrate or placebo was given per day, was followed by a period of 3 weeks, in which an oral dose of 10 g of creatine monohydrate or placebo was given per day [33]. It was evaluated whether oral creatine supplementation could attenuate the loss of muscle mass and malnutrition-inflammation score (MIS). MIS was developed to examine PEW in relation to inflammation, and is made up of 10 components, namely: weight change, dietary intake, gastro-intestinal symptoms, functional capacity, comorbidity, subcutaneous fat, signs of muscle wasting, body mass index (BMI), serum albumin level, and total iron-binding capacity (TIBC). Each of these 10 components of MIS has four levels for severity, ranging from 0 (normal) to 3 (severely abnormal). The sum of all 10 components can therefore range from 0 (normal) to 30 (severely malnourished and inflamed) [34]. Compared with the placebo arm, the creatine treated arm demonstrated a significant increase in lean body mass after 4 weeks of supplementation. Additionally, the creatine treated arm demonstrated a sizable reduction of MIS. However, to date, no studies have investigated whether creatine supplementation in patients with dialysis-dependent CKD is able to improve muscle strength, cognitive impairment, HRQoL, ability to perform daily tasks, infectious diseases, fatigue, or depression, individually or altogether. Furthermore, the aforementioned studies each used oral creatine supplementation with either single or multiple doses throughout the day, dissolved in relatively large volumes of water, which may negatively affect both patient compliance and fluid balance.

To avoid problems with compliance and fluid balance, and, furthermore, to prevent intradialytic losses of creatine to the dialysate, we aim to investigate the potential of intradialytic creatine supplementation in improving muscle status and HRQoL. To do so, the optimal creatine concentration in the dialysate in relation to maximal creatine uptake, as well as the tolerability of the intradialytic creatine application, must be determined first. The second goal of the study is to determine the effects of intradialytic creatine supplementation of chronic hemodialysis patients on muscle mass, muscle strength, cognitive functions, HRQoL, frailty, fatigue, and depression parameters.

## 3. A Clinical Pilot Study

### 3.1. Study Design and Setting

In this randomized, double-blind, placebo-controlled pilot study, a total of 16 hemodialysis patients will be included, which will be divided into four groups (0.5 mM, 1.0 mM, 1.5 mM, and 2.0 mM) each consisting of three patients receiving creatine and one receiving placebo. The study is a pilot study to generate data to allow for sample size calculations for a future larger study to be performed, as no data on the standard deviations of changes of intra-erythrocyte creatine concentrations over time and other outcome parameters over time are currently available to allow for an adequate sample size calculation. So, no sample size was calculated for the current study. The study will be conducted at the University Medical Center Groningen (UMCG) and the Dialysis Center Groningen (DCG). The protocol was written in accordance with the Standard Protocol Items: Recommendations for Interventional Trials (SPIRIT) guideline. Ethics committee approval will be obtained from the UMCG, the Netherlands.

The study population comprises of patients with dialysis-dependent CKD being treated with hemodialysis. The targeted study population of dialysis-dependent kidney disease patients in the local study centers is primarily >55 years old, predominantly Caucasian, with similar proportions of men and women, with approximately 50% of patients having residual diuresis. Only subjects who provide written informed consent are eligible. Additional inclusion criteria for this study are as follows:

- Age ≥ 18 years;
- Hemodialysis treatment in the UMCG or DCG;
- Dialysis vintage ≥2 months;
- Conventional hemodialysis, thrice weekly treatment with three to five hours per dialysis treatment;
- Hemoglobin at previous routine monthly assessment greater than or equal to 6.5 mmol/L;
- Signed informed consent.
- Exclusion criteria for the current study are:
- Pregnancy;
- Presence of clinical signs of infection;
- Confirmed diagnosis of malignancies;
- Incapacity of the Dutch language;
- Inability to complete questionnaires.

### 3.2. Interventions, Blinding, and Randomization

**Figure 2:**
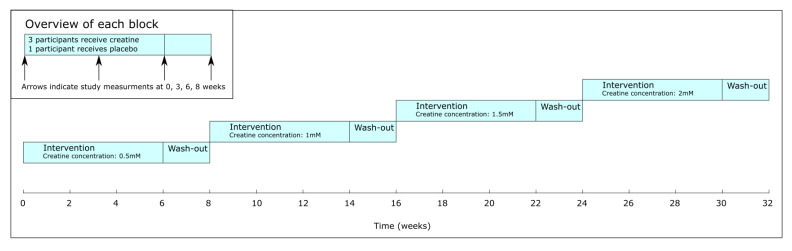
Timeline overview.

In this study, creatine will be added to the dialysis fluid. Adverse events are defined as any untoward medical occurrence in patients participating in the study. These adverse events do not necessarily have to have a causal relationship with the treatment. No major side-effects are expected. As the principle of intradialytic creatine supplementation is new, this pilot study will take a step-up approach with block randomization. The hemodialysis patients are divided into four different dosage groups in which patients will continuously receive either 0.5 mM, 1.0 mM, 1.5 mM, or 2.0 mM of creatine dissolved into the dialysis fluid during the entirety of a dialysis session. Each group consisted of four patients, in which three patients will be randomized to receive creatine and one patient received the placebo. Randomization will be performed according to the subsequent blocks, each of which will consiste of three patients receiving creatine and one receiving placebo. The first randomization block will be assigned to a creatine dosage of 0.5 mM. If after one week no adverse effects are observed, the next, higher dosage group will be started (Figure 2).

Oral supplementation has been extensively studied in the last thirty years, both in athletes as well as in the general population, in various age groups ranging from children to the elderly [35,36]. Oral supplementation with chemically pure creatine monohydrate, as used in this study, if taken within the officially recommended dosages, is safe with no significant side effects, except for moderate weight gain [33,37]. This weight gain is initially due to water retention, and in the long term is due to an increase in fat free muscle mass [33]. In dialysis patients, the initial water retention can be compensated by the dialysis itself. The increase in fat free muscle mass would, in fact, be beneficial for dialysis-dependent CKD, as they often suffer from PEW and/or sarcopenia. On top of that, as the gastrointestinal tract is spared when using intradialytic supplementation, complaints such as gastrointestinal complaints and bloating are unlikely to occur.

The patients will receive creatine supplementation or placebo (hemodialysis quality water) during each hemodialysis session for a total length of 6 weeks. Creatine-monohydrate, Creapure^®^ “Pharma Quality” (not GMP), produced by AlzChem Trostberg, Germany will be used for the preparation of a 50 mmol/L stock solution of creatine which will be added to the dialysis fluid to reach the projected dialysate concentrations, as indicated above.

To ensure the double-blind design, optically similar solutions will be packed and coded by Ace Pharmaceuticals, the Netherlands. The creatine solution will be added with an infusion pump at a sampling tip on the afferent dialysis fluid hose for the artificial kidney. Depending on the setting of the infusion pump and the dialysis flow, different creatine concentrations (between 0.5 and 2.0 mM) will be reached. The total dialysis treatment duration for the study was six weeks, with three dialysis sessions per week. The total creatine uptake at the different dosages (0.5 mM, 1.0 mM, 1.5 mM, or 2.0 mM) will be calculated as the added amount of creatine minus the creatine measured in the collected hemodialysis fluid (the creatine that did not diffuse to the blood).

### 3.3. Measurements

Study measurements will be performed at baseline, week 3, week 6, and after a washout period of 2 weeks. The primary outcome parameters of the study will be the assessment of the plasma creatine and intra-erythrocyte creatine concentration. At each study visit, the blood, interdialytic urine, and dialysate will be collected from each participant. Blood will be drawn from the hemodialysis lines both prior to and after the hemodialysis treatment. Participants with residual diuresis will be instructed to collect all of the urine in the interdialytic interval prior to the hemodialysis session of the visit, according to a strict protocol. At the end of the hemodialysis session prior to the hemodialysis session of the study visit, participants will be instructed to start collecting all of the urine until the start of the hemodialysis session of the study visit. For participants dialyzing thrice weekly, this will be roughly 48 h. During the hemodialysis session, all dialysate will be collected in a 200-L tank. The total dialysate volume will be measured by calculating the weight difference of the tank before and after the hemodialysis session. At the end of hemodialysis, all dialysate will be homogenized, and samples will be taken for analyses and storage.

Plasma, intra-erythrocyte, and urinary creatine concentrations will be measured with liquid chromatography mass spectrometry (LC-MS/MS), validated according to ISO 151 89 guidelines, using an LC30AD UPLC (Shimadzu, Kyoto, Japan) and an API-4500 mass spectrometer (SCIEX, Framingham, MA, USA). Creatine will be separated using a Phenomenex Kinetex Evo™ C18 (2.6 µm, 100 Å, 150 × 4.6 mm) column, with a Phenomenex SecurityGuard™ ULTRA cartridges for C18 (2.6 µm, 2 × 4.6 mm) guard column. Creatine is detected using positive-ion electrospray ionization in the multiple reaction monitoring mode using the following transitions: m/z 132→90 for creatine and m/z 135→93 for the internal standard (D3-creatine). Data will be analyzed using Analyst MD 1.6.2 (Sciex). The intra-assay and inter-assay coefficients of variation are 3.9% and 6.5% at 17 µmol/L and 1.7% and 1.7% at 1065 µmol/L, respectively.

Anthropometry measurements will include body weight in kilograms (kg), body height in centimeters (cm), and hip and waist circumference both in centimeters (cm). Body weight will be measured both before and after the hemodialysis session in lightweight clothing without shoes using a calibrated digital measuring scale (seca 877, seca, Hamburg, Germany). Body height will be measured before the start of the hemodialysis session using a wall-secured stadiometer (seca 222). Waist and hip circumference will be measured in centimeters (cm) twice after the hemodialysis session, using a measuring tape roll with a standardized retraction mechanism (seca 201). Blood pressure (mmHg) will be measured according to a standard clinical protocol, using an automatic device (Phillips Suresign VS2+, Andover, MA, USA). Blood pressure (mmHg) will be measured before the start of the hemodialysis session, each hour during the dialysis period, and at the end of the hemodialysis session. Blood pressure will be measured in standing position before and after the dialysis session. Patients will be asked to stand up straight for 1 min, after which blood pressure and heart rate measurements will be performed. Body composition will be determined using a multifrequency bioelectrical impedance analyzer (BIA, Quadscan 4000, Bodystat, Douglas, UK) at 5, 50, 100, and 200 Hz, which allowes for distinguishing between lean body mass and fat body mass, taking into account the differences in volume status [38]. According to recommendations [39], the BIA measurement will be performed before the hemodialysis session, with the participant in a supine position with arms and legs abducted from the body.

Hand grip strength will be assessed in kilograms (kg) in both arms with the Jamar Hydraulic Hand Dynamometer (Patterson Medical JAMAR 5030J1, Warenville, IL, USA) [40]. The hand grip strength will be assessed before the start of the hemodialysis session, in a seated position, with shoulders in adduction, arms rotated into a neutral position, elbows flexed to 90°, and forearms and wrists held in a neutral position. After the participant is positioned in the right way, they will be instructed to perform a maximal isometric contraction. To avoid the occurrence of motivation bias, all patients will be encouraged through vocal commands at high volume [41]. Hand grip strength will be tested three times with an interval of 30 s between each attempt. As it has been shown that the difference between the arm with the fistulae compared with the contralateral is greater than the difference between the dominant compared with the non-dominant hand [42], the measurements will be performed on the arm in which no fistula is present, and when no fistula is present in either of the arms, measurements will be performed on the hand of the dominant arm.

The Short Physical Performance Battery (SPPB) is a relatively simple test that can be used to gain insight into balance, gait, strength, and endurance [43], and will be performed before the start of the hemodialysis session. The SPPB consists of three separate tests, namely: a balance test, a walking test, and a repeated chair–stand test. For the balance test, participants need to stand still for at least 10 s in three progressively difficult positions, with the feet together in side-by-side, semi-tandem, and tandem positions. Lower limb strength will be tested with the repeated chair–stand test. Participants will be asked to fold their arm across their chests while standing up and sitting down five times as quickly as possible, and the time will be recorded in seconds (s).

The 4 m Gait Speed Test (4MWT) will be used to measure locomotion [44,45,46]. For the 4MWT, two pylons will be placed 4 m apart and the subject will be instructed to walk at their usual place. Time from start to end of the 4 m will be recorded in seconds (s). The 4MWT will be measured twice, before the start of the hemodialysis session.

Forced expiratory volume for 1 s (FEV_1_) will be measured in liters (L), both before the start of the dialysis session as well as at the end of the hemodialysis session, by means of an Asma-1 handheld spirometer (Vitalograph, Buckingham, UK) [47].

The 9-Hole Peg Test (9-HPT, Sammsons Preston Rolyan, Chicaco, IL, USA) requires participants to place and remove nine pegs into nine holes, one at a time, as quickly as possible, and is therefore a measure of hand dexterity [48]. Participants will be asked to perform the 9-HPT with the dominant hand first and then with the non-dominant hand.

The following questionnaires will be administered: Six-Dimensional EuroQol Instrument (EQ-6D) [49] and the Short Form 36 Health Survey (SF36) [50] to assess HRQoL; Checklist Individual Strength (CIS) [51] to assess fatigue; Dialysis Symptom Indicator (DSI) [16] to assess the prevalence, severity, and impact of symptoms in hemodialysis patients; Groninger Frailty Indicator (GFI) [52] to identify frailty; Cognitive Failure Questionnaire (CFQ) [53] to assess cognitive failure; Patient Health Questionnaire-9 (PHQ-9) [54] to measure depressive disorders and depression severity; and the Patient-Generated Subjective Global Assessment (PG-SGA) [55,56,57] to assess nutritional status. Patients can fill in the questionnaires at home before the start of the hemodialysis session, or during the dialysis session with the help of a researcher.

### 3.4. Outcomes

The aim of the pilot study is to explore the creatine uptake in the circulation and tissues following different creatine supplementation dosages. Therefore, the main parameters for the pilot study are the plasma creatine concentration and intra-erythrocytic creatine concentration of both the pre- and post-hemodialysis samples. Intra-erythrocytic creatine concentration will be used as a non-invasive proxy for creatine tissue uptake. The secondary study parameters are hand grip strength as a measure of muscle strength, the combined interdialytic urinary and intradialytic dialysate excretion of creatinine as a measure of muscle mass [58], and bioelectrical impedance analysis (BIA) as a measure of body composition. Other study parameters are N-terminal pro-brain natriuretic peptide (NT-proBNP) as a cardiac function marker; high sensitivity troponin T (hs-TNT) as a cardiac ischaemia marker; C-reactive protein as an inflammation marker; self-reported physical health using the EQ-6D, SF36, and DSI; fatigue using the CIS; and cognitive functions using the CFQ.

### 3.5. Statistical Analyses

Statistical analyses will be performed using R statistical software (Vienna, Austria) (http://cran.r-project.org/ (accessed on 1 August 2021)). The results will be expressed as mean ± standard deviation (SD), median (interquartile range), or number (percentage) for normally distributed, skewed, and categorical data, respectively. For all of the analyses, *p*-value < 0.05 will be considered statistically significant. To determine the effect of creatine supplementation on the primary and secondary outcomes, linear mixed-effect models will be used to compare the change in outcome over time between the different treatment groups. Linear mixed-effect models will be performed using the “lmer” function of the “lme4” package. Treatment group, time, and the interaction between treatment group and time will be used as fixed factors, and random intercepts and slopes will be added for the subjects. To minimize the false positive rate, as recommended by Barr et al., we aim to use the maximal random effect structure, even if a random effect does not improve the overall model fit [59].

## 4. Discussion

Creatine supplementation as a nutritional intervention has been extensively studied in several populations, showing promising results [60,61,62]. To date, almost all creatine supplementation studies have been performed with oral supplementation. In dialysis-dependent kidney disease patients, the oral supplementation of creatine is less suitable as it requires dissolving the creatine in large volumes of water, which may negatively affect the fluid balance. In addition, the causes for creatine deficiency in dialysis-dependent kidney disease patients, being impaired endogenous synthesis [4,7], losses to the dialysis fluid [17,18], and insufficient intake [19,20], are chronic in nature and therefore require long-term supplementation of creatine. As many CKD patients are dependent on dialysis for years, sometimes even lifelong, treating creatine deficiency with oral supplementation requires a high level of compliance. In addition, oral supplementation does not prevent losses of creatine to the dialysis fluid. In contrast to oral supplementation, intradialytic supplementation offers the possibility to supplement creatine in a controlled manner, while preventing unopposed losses of creatine to the dialysis fluid, volume overload due to the necessary ingestion of large volumes of water, and potential problems with compliance.

A creatine deficient state is not without consequences. It has become increasingly clear that low creatine levels play an important role in many different causes for impaired HRQoL and have higher mortality rates in hemodialysis patients. For example, a recent study showed that higher plasma creatine concentrations are associated with lower odds of low muscle mass, low protein intake, hypoalbuminemia, and severe fatigue, indicating a potential role for creatine supplementation in hemodialysis patients [18].

Protein-energy-wasting (PEW), a progressive depletion of protein and energy stores, is highly prevalent in hemodialysis patients (up to 50–75%) and is associated with both increased morbidity and mortality, and impaired quality of life. Potential causes of PEW in patients with dialysis-dependent CKD include reduced protein and energy intake, reduced physical activity, increased catabolism, reduced anabolism, and comorbidities (e.g., diabetes), as well as the dialysis treatment itself, causing, among others, a loss of amino acids to the dialysate [63,64]. Additionally, the situation is exacerbated by inflammatory processes, and loss of residual renal function [63]. These causes often occur simultaneously and exacerbate the general pathological state of dialysis patients.

It has been shown that oral creatine supplementation increases the intramuscular total creatine (creatine plus phosphocreatine) concentrations [62] and, in parallel, significantly increases muscle mass [65] and muscle performance for high-intensity and endurance performance [62,66]. In addition, there is a growing body of evidence that creatine supplementation, combined with moderate resistance training, can, at least partially, counteract the loss of muscle mass caused by ageing or immobilization [67,68].

Furthermore, hemodialysis patients frequently suffer from fatigue [2,69]. The burden of fatigue is underscored by the results of a cross-sectional study, in which 94% of the patients would accept more frequent hemodialysis if it would increase their energy level, but only 19% would do so for an increase in survival time of up to 3 years [70]. It has been hypothesized previously that creatine administration plays an important role in reducing both mental and muscular fatigue by increasing the brain content of phosphocreatine [71]. Therefore, intradialytic creatine supplementation may benefit hemodialysis patients by potentially attenuating the fatigue often experienced with kidney disease.

Another contributor to increased mortality and impaired HRQoL in hemodialysis patients is cognitive impairment [72]. Cognitive impairment is associated with lower compliance concerning nutritional restrictions, fluid restrictions, and medication [73]. The underlying pathogenesis is not fully understood, but it has been indicated that creatine supplementation improves brain health, improves cognition, and is effective at alleviating brain ischemia and hypoxia [30]. Importantly, creatine has also been shown to alleviate treatment-resistant depression, especially in women [74,75,76], and recent study results indicate a significant negative relationship between dietary creatine intake and depression in a nationally representative adult cohort in the USA, indicating that a low creatine intake may enhance the incidence of depression [75]. Therefore, intradialytic creatine supplementation may benefit hemodialysis patients by potentially improving the mental complaints often experienced with kidney disease.

Additionally, as creatine has a positive impact on both the development and activation of the innate and adaptive immune response [31], dialysis patients who are supplemented with creatine may have a lower incidence of infections during the time of chronic dialysis treatments.

Furthermore, as cardiovascular diseases are prominent pathologies in dialysis patients, creatine, with its reported benefit for vascular health, such as alleviating oxidative stress and inflammation [71,77,78], may be helpful in these respects in this vulnerable population.

During hemodialysis treatment, erythrocytes are subject to mechanical and oxidative stress, potentially leading to anemia. Studies suggest that creatine, by its ability to inhibit erythrocyte lipid peroxidation, may contribute to the maintenance of normal cell deformability [79,80,81]. Intradialytic creatine supplementation could potentially lead to reduced losses of erythrocytes, and may therefore reduce the erythropoietin (EPO) requirements of the dialysis patients. The reduced administration of EPO will circumvent the possible EPO-supported progression of tumors and should markedly reduce costs to the healthcare system [82,83].

## 5. Conclusions

Patients with CKD relying on dialysis treatments likely suffer from creatine deficiency due to a decreased endogenous production of creatine and unopposed losses of creatine from the blood into the dialysate. In the current study, we have provided the rationale for intradialytic creatine supplementation and described the study protocol for a pilot study in preparation for a large double-blind, placebo-controlled supplementation trial.

Intradialytic creatine supplementation may help to maintain creatine homeostasis among dialysis-dependent CKD patients, and consequently improve important causes for impaired HRQoL, including protein energy wasting (PEW), fatigue, muscle weakness, depression, and cognitive impairment.

## Abbreviations

AGA—arginine:glycine amidino-transferase; AMPK—AMP-activated protein kinase; ATP—adenosine-tri-phosphate; Cr—creatine; PCr—Phosphocreatine; CK—creatine kinase; ESKD—end-stage kidney disease; GAA—guanidinoacetate; GAMT—guanidino-acetate-amino-transferase; HRQoL—health-related quality of life.
